# Short Sleep Duration Among Infants, Children, and Adolescents Aged 4 Months–17 Years — United States, 2016–2018

**DOI:** 10.15585/mmwr.mm7038a1

**Published:** 2021-09-24

**Authors:** Anne G. Wheaton, Angelika H. Claussen

**Affiliations:** ^1^Division of Population Health, National Center for Chronic Disease Prevention and Health Promotion, CDC; ^2^Division of Human Development and Disabilities, National Center on Birth Defects and Developmental Disabilities, CDC.

Infants, children, and adolescents who do not get sufficient sleep are at increased risk for injuries, obesity, type 2 diabetes, poor mental health, attention and behavior problems, and poor cognitive development (*1*). The American Academy of Sleep Medicine (AASM) provides age-specific sleep duration recommendations to promote optimal health (*1*). CDC analyzed data from the 2016–2018 National Survey of Children’s Health (NSCH) to assess the prevalence of short sleep duration among persons in the United States aged 4 months–17 years. Overall, on the basis of parent report, 34.9% of persons aged 4 months–17 years slept less than recommended for their age. The prevalence of short sleep duration was higher in southeastern states and among racial and ethnic minority groups, persons with low socioeconomic status, and those with special health care needs. The prevalence of short sleep duration ranged from 31.2% among adolescents aged 13–17 years to 40.3% among infants aged 4–11 months. Persons aged 4 months–17 years with a regular bedtime were more likely to get enough sleep. Public health practitioners, educators, and clinicians might advise parents on the importance of meeting recommended sleep duration and implementing a consistent bedtime for healthy development.

NSCH is a population-based, nationally representative online and paper survey of parents or primary caregivers (parents) of noninstitutionalized U.S. persons aged ≤17 years. The survey is conducted by the U.S. Census Bureau under the direction of the Health Resources and Services Administration’s Maternal and Child Health Bureau.* NSCH asks parents about the physical and emotional health of one person aged ≤17 years selected at random from the household, as well as health care access and family characteristics. The weighted overall response rates were 40.7% in 2016, 37.4% in 2017, and 43.1% in 2018.^†^ Sleep duration questions were “During the past week, how many hours of sleep did this child get on an average day (count both nighttime sleep and naps)?”^§^ for infants and children aged 0–5 years, and for children and adolescents aged 6–17 years, “During the past week, how many hours of sleep did this child get on an average weeknight?”^¶^ On the basis of AASM recommendations (*1*), short sleep duration was defined as <12 hours for children aged 4–11 months, <11 hours for children aged 1–2 years, <10 hours for children aged 3–5 years, <9 hours for children aged 6–12 years, and <8 hours for adolescents aged 13–17 years. The bedtime question for all ages was “How often does this child go to bed at about the same time on weeknights?”** Regular bedtime was defined as a response of “always.” The study included 99,842 persons aged 4 months–17 years^††^ with responses to the sleep duration question (48,748 in 2016, 21,124 in 2017, and 29,970 in 2018).

Prevalence and 95% confidence interval (CI) of short sleep duration and regular bedtime were calculated for persons aged 4 months–17 years overall, by age group, by state, and by selected characteristics of the child and parent. Pairwise differences by sex, race/ethnicity, special health care needs status, overall health status, and regular bedtime were determined using t-tests. Tests for linear trend were conducted for family income level^§§^ and parent education level. P-values <0.05 were considered statistically significant. Analyses accounted for weighting^¶¶^ of the data and for the complex sampling design. SAS-callable SUDAAN (version 11.0.3; RTI International) was used to conduct all analyses. This activity was reviewed by CDC and was conducted consistent with applicable federal law and CDC policy.***

Overall, 34.9% of persons aged 4 months–17 years slept less than recommended for their age (Table 1). The prevalence of short sleep duration ranged from 31.2% among adolescents aged 13–17 years to 40.3% among infants aged 4–11 months. The percentage of persons aged 4 months–17 years with a regular bedtime was 33.9% overall and ranged from 23.8% among adolescents to 43.5% among infants. Among children aged 4 months–17 years, the prevalence of short sleep duration was lowest among non-Hispanic White children (28.8%) and higher among non-Hispanic Black children (50.8%) than among Hispanic children (39.1%) or non-Hispanic children of any other race (other race) (34.6%) (Table 2). The prevalence decreased with increasing family income level and parent educational attainment. The prevalence of short sleep duration was higher among persons aged 4 months–17 years with special health care needs than among those without special health care needs (38.3% versus 34.1%); and among those with good, fair, or poor health than among those with excellent or very good health (46.8% versus 33.5%). Overall, the prevalence of short sleep duration was lower among persons aged 4 months–17 years with a regular bedtime than among those without a regular bedtime (27.5% versus 38.6%). Similar patterns were observed within each age group.

**TABLE 1:** Prevalence of short sleep duration* and regular bedtime^†^ among persons aged 4 months–17 years, by age group — National Survey of Children’s Health, United States, 2016–2018

Characteristic	% (95% CI)^§^					
	All persons aged 4 mos–17 yrs (n = 99,842)	Infants aged 4–11 mos (n = 2,499)	Children aged 1–2 yrs (n = 10,147)	Children aged 3–5 yrs (n = 15,290)	Children aged 6–12 yrs (n = 36,179)	Adolescents aged 13–17 yrs (n = 35,727)
Short sleep duration	34.9 (34.2–35.6)	40.3 (35.9–44.7)	33.3 (31.2–35.4)	34.8 (33.1–36.7)	37.4 (36.3–38.6)	31.2 (30.1–32.4)
Regular bedtime	33.9 (33.2–34.6)	43.5 (39.1–47.9)	40.9 (38.7–43.0)	37.3 (35.5–39.1)	37.0 (35.9–38.2)	23.8 (22.7–24.9)

**Abbreviation:** CI = confidence interval.

* Short sleep duration is defined as <12 hours for infants aged 4–11 months, <11 hours for children aged 1–2 years, <10 hours for children aged 3–5 years, <9 hours for children aged 6–12 years, and <8 hours for adolescents aged 13–17 years.

^†^ Regular bedtime is defined as a response of “always” to the question about how often the person aged <18 years goes to bed at approximately the same time on weeknights.

^§^ Weighted percentages. The National Survey of Children’s Health is weighted to be representative of the U.S. population of noninstitutionalized persons aged ≤17 years. https://www2.census.gov/programs-surveys/nsch/technical-documentation/methodology/NSCH-Guide-to-Multi-Year-Estimates.pdf

**TABLE 2:** Prevalence of short sleep duration* among persons aged 4 months–17 years, by age group and selected characteristics — National Survey of Children’s Health, United States, 2016–2018

Characteristic	% (95% CI)^†^					
	All persons aged 4 mos–17 yrs	Infants aged 4–11 mos	Children aged 1–2 yrs	Children aged 3–5 yrs	Children aged 6–12 yrs	Adolescents aged 13–17 yrs
**Sex**						
Male	35.0 (34.0–36.0)	40.6 (34.4–47.0)	32.8 (29.9–35.7)	35.2 (32.8–37.7)	38.4 (36.8–40.0)	30.4 (28.9–32.1)
Female	34.7 (33.7–35.7)	39.9 (34.0–46.1)	33.8 (30.8–36.8)	34.5 (31.9–37.1)	36.4 (34.8–38.1)	32.1 (30.3–33.8)
**Race/Ethnicity**						
Hispanic	39.1 (37.1–41.1)	41.5 (29.9–54.1)^§^	40.5 (34.6–46.7)	42.4 (37.3–47.7)	43.3 (40.1–46.6)	30.3 (27.1–33.7)
White, non-Hispanic	28.8 (28.1–29.5)	32.9 (28.2–38.0)	26.1 (24.2–28.2)	27.9 (26.2–29.7)	30.1 (29.0–31.1)	28.1 (27.1–29.2)
Black, non-Hispanic	50.8 (48.6–53.0)	64.2 (51.1–75.5)^§^	50.2 (42.7–57.7)	51.7 (46.1–57.4)	56.4 (53.0–59.8)	42.2 (38.5–46.0)
Other,^¶^ non-Hispanic	34.6 (32.9–36.4)	46.8 (36.8–57.2)^§^	35.6 (29.7–41.9)	32.3 (28.7–36.1)	34.8 (32.1–37.6)	33.4 (30.5–36.5)
Family income level**						
FPL<100%	45.6 (43.6–47.7)	54.2 (41.5–66.4)^§^	45.4 (39.5–51.5)	49.2 (44.1–54.3)	51.6 (48.4–54.8)	33.4 (30.1–36.9)
100%≤FPL<200%	39.2 (37.3–41.2)	49.9 (39.6–60.2)^§^	39.1 (33.6–44.8)	41.4 (36.4–46.5)	41.1 (38.1–44.1)	34.0 (30.9–37.2)
200%≤FPL<400%	33.2 (31.9–34.6)	33.3 (25.3–42.4)	32.7 (28.3–37.3)	32.7 (29.4–36.2)	35.8 (33.7–37.9)	30.4 (28.3–32.5)
FPL≥400%	26.0 (25.0–27.0)	29.2 (23.7–35.5)	21.9 (18.9–25.4)	22.5 (20.1–25.0)	26.3 (24.8–27.7)	28.7 (27.1–30.5)
**Parent education**						
Less than high school	46.2 (42.4–50.0)	60.3 (34.7–81.2)^§^	39.7 (27.8–53.0)^§^	54.0 (43.1–64.5)^§^	53.7 (47.7–59.5)	34.1 (28.6–40.1)
High school	43.3 (41.4–45.2)	54.1 (43.8–64.1)^§^	49.0 (42.7–55.4)	47.4 (42.2–52.7)	46.3 (43.3–49.3)	34.2 (31.2–37.3)
Some college	39.3 (37.8–40.7)	49.2 (40.3–58.2)	39.2 (34.7–44.0)	40.6 (36.8–44.4)	43.0 (40.7–45.3)	32.2 (30.0–34.5)
College graduate	27.2 (26.5–28.0)	28.6 (24.3–33.3)	24.9 (22.6–27.3)	25.0 (23.2–26.8)	27.8 (26.6–29.0)	28.7 (27.4–30.1)
**Special health care needs^††^**						
Yes	38.3 (36.8–39.7)	30.5 (19.5–44.2)^§^	30.9 (25.3–37.1)	38.3 (34.3–42.6)	42.8 (40.7–45.1)	33.7 (31.4–36.1)
No	34.1 (33.3–34.9)	40.7 (36.2–45.3)	33.5 (31.3–35.7)	34.3 (32.3–36.3)	35.9 (34.6–37.3)	30.4 (29.1–31.8)
**Health status**						
Excellent or very good	33.5 (32.8–34.2)	39.4 (35.0–44.0)	32.4 (30.4–34.5)	33.8 (31.9–35.6)	35.3 (34.2–36.5)	30.3 (29.1–31.5)
Good, fair, or poor	46.8 (44.1–49.5)	59.8 (40.2–76.7)^§^	45.4 (35.1–56.1)^§^	45.6 (38.1–53.3)	54.6 (50.5–58.7)	38.0 (33.8–42.4)
**Regular bedtime** ^§§^						
Yes	27.5 (26.3–28.9)	34.6 (27.6–42.3)	28.5 (25.5–31.7)	29.1 (25.8–32.6)	30.5 (28.6–32.6)	17.4 (15.5–19.6)
No	38.6 (37.8–39.4)	44.8 (39.5–50.2)	36.6 (33.8–39.4)	38.1 (36.0–40.2)	41.4 (40.0–42.8)	35.6 (34.2–37.0)
**State**						
Alabama	42.5 (39.3–45.8)	46.7 (28.3–66.2)^§^	41.7 (32.0–52.0)	39.2 (32.1–46.8)	49.6 (44.3–54.9)	34.4 (29.3–39.9)
Alaska	27.7 (24.7–30.8)	32.1 (19.6–47.8)^§^	28.3 (20.8–37.3)	26.6 (20.1–34.2)	31.9 (26.9–37.4)	20.5 (16.2–25.8)
Arizona	35.6 (32.3–38.9)	27.1 (13.8–46.2)^§^	36.7 (27.1–47.4)^§^	35.9 (28.1–44.5)	38.1 (32.9–43.6)	32.2 (26.8–38.0)
Arkansas	40.8 (37.4–44.2)	47.6 (31.5–64.1)^§^	35.7 (26.3–46.4)^§^	45.9 (37.7–54.4)	44.2 (38.8–49.7)	34.0 (28.2–40.4)
California	34.2 (30.9–37.6)	44.9 (24.2–67.6)^§^	30.3 (22.0–40.1)	33.8 (26.1–42.4)	38.2 (32.9–43.8)	28.5 (23.4–34.2)
Colorado	26.8 (24.1–29.7)	27.1 (15.7–42.6)^§^	25.8 (17.6–36.1)	28.2 (22.0–35.3)	26.9 (22.6–31.6)	26.3 (21.7–31.6)
Connecticut	32.8 (29.9–36.0)	46.5 (27.0–67.0)^§^	27.8 (19.0–38.6)	36.7 (28.5–45.7)	31.7 (27.1–36.7)	32.8 (28.2–37.8)
Delaware	39.1 (35.9–42.5)	46.2 (29.0–64.3)^§^	35.9 (26.4–46.6)^§^	42.4 (34.0–51.3)	42.1 (37.0–47.3)	33.5 (28.4–39.0)
District of Columbia	36.5 (32.8–40.3)	31.2 (17.9–48.6)^§^	36.7 (27.7–46.7)	25.9 (19.4–33.7)	44.5 (38.0–51.1)	34.7 (27.5–42.6)
Florida	38.6 (35.3–41.9)	50.0 (29.0–71.0)^§^	36.0 (27.5–45.6)	43.3 (34.6–52.5)	38.4 (33.4–43.6)	36.2 (30.5–42.2)
Georgia	40.3 (37.0–43.6)	38.9 (22.2–58.7)^§^	33.5 (24.2–44.3)^§^	39.4 (31.4–48.1)	47.1 (41.8–52.5)	32.9 (28.0–38.2)
Hawaii	38.4 (35.5–41.3)	41.6 (26.2–58.9)^§^	32.2 (23.9–41.7)	32.2 (25.6–39.6)	43.0 (38.3–47.9)	38.4 (33.6–43.5)
Idaho	26.9 (24.4–29.6)	22.0 (12.5–35.6)^§^	25.0 (18.5–32.8)	28.6 (21.9–36.4)	28.4 (24.3–32.9)	25.1 (21.2–29.5)
Illinois	30.1 (27.2–33.1)	53.4 (36.1–69.9)^§^	23.6 (16.5–32.6)	27.9 (20.7–36.4)	32.2 (27.5–37.2)	28.4 (23.7–33.5)
Indiana	38.6 (35.5–41.7)	36.7 (20.8–56.0)^§^	45.8 (36.6–55.3)	35.4 (28.3–43.2)	40.4 (35.3–45.6)	36.0 (31.0–41.3)
Iowa	29.0 (26.3–32.0)	28.7 (12.9–52.4)^§^	20.5 (13.3–30.3)	28.9 (22.6–36.2)	34.3 (29.6–39.4)	25.0 (21.3–29.2)
Kansas	30.3 (27.3–33.4)	39.9 (21.3–62.0)^§^	27.0 (18.0–38.5)^§^	31.0 (24.2–38.8)	32.5 (27.9–37.5)	26.7 (22.2–31.8)
Kentucky	35.8 (32.8–39.0)	28.9 (12.8–53.0)^§^	31.0 (23.2–40.2)	28.8 (22.3–36.3)	46.0 (40.6–51.5)	29.4 (25.1–34.3)
Louisiana	46.6 (43.2–49.9)	34.0 (19.7–51.9)^§^	44.9 (34.8–55.6)^§^	47.0 (39.3–54.9)	53.1 (47.7–58.5)	39.0 (33.5–44.9)
Maine	25.3 (22.7–28.1)	29.0 (14.1–50.3)^§^	24.9 (17.2–34.7)	20.4 (14.9–27.4)	25.6 (21.3–30.4)	27.8 (23.6–32.3)
Maryland	37.4 (34.3–40.7)	16.7 (6.9–35.0)^§^	36.7 (27.3–47.2)	43.5 (35.4–51.8)	41.8 (36.8–47.1)	30.5 (25.7–35.8)
Massachusetts	30.0 (27.1–33.1)	35.9 (21.1–54.0)^§^	25.7 (17.3–36.3)	30.7 (23.4–39.1)	30.9 (26.1–36.2)	29.5 (24.9–34.5)
Michigan	32.4 (29.5–35.6)	40.1 (22.0–61.3)^§^	29.7 (21.2–39.9)	34.5 (27.0–42.9)	33.9 (29.1–39.0)	29.6 (25.1–34.6)
Minnesota	25.4 (22.6–28.4)	37.9 (22.9–55.6)^§^	27.4 (17.8–39.7)^§^	27.3 (20.9–34.8)	26.2 (21.9–31.1)	20.6 (16.3–25.6)
Mississippi	48.9 (45.5–52.2)	52.6 (30.0–74.2)^§^	45.1 (35.9–54.7)	44.5 (36.6–52.6)	60.7 (55.5–65.7)	36.2 (30.8–42.0)
Missouri	35.8 (32.7–38.9)	24.0 (12.7–40.8)^§^	35.9 (26.8–46.1)	37.0 (29.8–44.8)	37.6 (32.5–42.9)	33.8 (29.1–38.8)
Montana	27.5 (24.7–30.5)	35.5 (20.6–53.9)^§^	30.4 (21.7–40.7)	29.1 (22.4–36.9)	28.0 (23.4–33.1)	23.5 (19.6–27.9)
Nebraska	30.8 (27.7–34.0)	49.8 (31.8–67.9)^§^	29.1 (21.3–38.3)	26.0 (19.8–33.2)	31.0 (26.3–36.2)	31.2 (25.6–37.4)
Nevada	32.4 (29.2–35.6)	33.1 (19.0–51.2)^§^	33.4 (24.1–44.3)^§^	28.9 (22.1–36.8)	35.9 (30.8–41.3)	29.1 (23.7–35.1)
New Hampshire	27.0 (24.5–29.7)	28.9 (15.9–46.7)^§^	23.1 (14.4–34.8)^§^	23.4 (17.7–30.3)	26.4 (22.5–30.6)	30.8 (26.7–35.4)
New Jersey	34.6 (31.5–37.9)	41.4 (21.3–64.8) ^§^	34.8 (24.8–46.3)^§^	37.7 (30.2–45.9)	33.0 (28.0–38.3)	34.2 (29.1–39.6)
New Mexico	33.8 (30.4–37.2)	42.5 (23.9–63.5)^§^	34.5 (24.8–45.8)^§^	35.9 (28.1–44.5)	35.7 (30.3–41.5)	28.6 (23.2–34.6)
New York	36.2 (32.9–39.6)	39.5 (22.0–60.2)^§^	25.5 (17.5–35.4)	32.2 (24.7–40.8)	39.7 (34.4–45.3)	37.4 (31.7–43.5)
North Carolina	36.8 (33.5–40.3)	48.6 (31.2–66.2)^§^	36.5 (26.3–48.0)^§^	38.9 (31.2–47.3)	37.7 (32.4–43.4)	33.0 (27.6–38.9)
North Dakota	25.4 (22.6–28.4)	24.1 (13.3–39.8)^§^	33.4 (24.4–43.8)	24.1 (17.7–31.8)	24.6 (20.2–29.6)	23.2 (19.3–27.7)
Ohio	34.8 (31.8–38.0)	44.3 (24.8–65.7)^§^	32.9 (23.8–43.5)	26.3 (20.2–33.4)	38.7 (33.7–43.9)	34.2 (29.0–39.8)
Oklahoma	35.3 (32.3–38.5)	37.1 (20.7–57.1)^§^	42.3 (32.5–52.6)^§^	30.6 (23.9–38.2)	36.0 (31.3–40.9)	34.2 (29.0–39.8)
Oregon	27.6 (24.6–30.7)	31.9 (18.0–50.1)^§^	33.5 (23.9–44.7)^§^	35.8 (27.7–44.8)	23.9 (19.7–28.7)	25.3 (20.8–30.5)
Pennsylvania	32.8 (29.9–35.9)	40.9 (24.3–59.8)^§^	27.5 (20.4–36.0)	32.9 (25.6–41.2)	35.9 (31.0–41.0)	29.0 (24.5–33.9)
Rhode Island	33.9 (30.8–37.1)	58.2 (40.0–74.4)^§^	25.5 (18.4–34.2)	30.2 (22.9–38.6)	35.5 (30.6–40.7)	33.7 (28.3–39.5)
South Carolina	40.2 (37.0–43.5)	30.6 (12.8–57.1)^§^	45.6 (36.2–55.4)	41.8 (33.5–50.5)	41.7 (36.6–47.0)	35.5 (30.2–41.1)
South Dakota	30.0 (27.2–32.9)	46.8 (33.0–61.2)^§^	29.1 (22.3–37.0)	31.3 (24.4–39.1)	28.9 (24.4–33.9)	27.7 (23.1–32.8)
Tennessee	39.1 (36.0–42.4)	58.3 (39.8–74.7)^§^	41.5 (32.4–51.1)	39.7 (32.4–47.6)	42.5 (37.4–47.7)	30.2 (25.5–35.4)
Texas	36.7 (33.3–40.2)	35.3 (21.1–52.6)^§^	41.8 (32.0–52.4)^§^	40.4 (31.9–49.6)	38.0 (32.6–43.8)	30.7 (25.0–37.1)
Utah	29.3 (26.5–32.3)	25.9 (14.0–42.8)^§^	31.6 (22.6–42.4)	24.0 (18.6–30.3)	32.3 (27.7–37.3)	28.0 (23.1–33.5)
Vermont	25.6 (22.9–28.6)	47.5 (29.4–66.2)^§^	26.5 (19.0–35.5)	26.6 (19.8–34.7)	25.6 (21.3–30.5)	21.8 (17.9–26.2)
Virginia	32.7 (29.7–35.8)	45.1 (26.0–65.7)^§^	33.1 (24.0–43.6)	32.2 (25.0–40.4)	35.1 (30.3–40.3)	28.5 (24.0–33.4)
Washington	30.5 (27.5–33.7)	43.4 (26.6–61.9)^§^	32.4 (23.4–42.9)	30.4 (23.6–38.3)	31.3 (26.3–36.7)	27.1 (22.1–32.7)
West Virginia	42.9 (39.7–46.3)	45.2 (30.0–61.2)^§^	47.4 (36.3–58.8)^§^	46.2 (38.3–54.3)	42.9 (37.9–48.1)	38.8 (33.4–44.5)
Wisconsin	28.9 (26.2–31.9)	46.3 (28.0–65.8)^§^	26.5 (19.0–35.8)	23.1 (17.3–30.3)	30.6 (26.3–35.4)	28.5 (24.0–33.4)
Wyoming	31.9 (28.9–35.0)	32.3 (18.0–50.8)^§^	33.6 (25.6–42.7)	30.2 (23.1–38.3)	36.4 (31.5–41.7)	25.1 (20.6–30.1)

**Abbreviations:** CI = confidence interval; FPL = federal poverty level.

* Short sleep duration is defined as <12 hours for infants aged 4–11 months, <11 hours for children aged 1–2 years, <10 hours for children aged 3–5 years, <9 hours for children aged 6–12 years, and <8 hours for adolescents aged 13–17 years.

^†^ Weighted percentages. Weighted estimates generalize to state and national resident populations. https://www2.census.gov/programs-surveys/nsch/technical-documentation/methodology/NSCH-Guide-to-Multi-Year-Estimates.pdf; https://www.census.gov/content/dam/Census/programs-surveys/nsch/tech-documentation/methodology/2016-NSCH-Methodology-Report.pdf; https://www.census.gov/content/dam/Census/programs-surveys/nsch/tech-documentation/methodology/2017-NSCH-Methodology-Report.pdf; https://www2.census.gov/programs-surveys/nsch/technical-documentation/methodology/2018-NSCH-Methodology-Report.pdf

^§^ Estimate might be unreliable. The absolute CI width is >20% or the relative CI width is >120% (1.2 times the estimate). https://www.childhealthdata.org/docs/default-source/drc/nsch_data-supression-and-display_revised_03-01-19.pdf

^¶^ Includes American Indian or Alaska Native, Native Hawaiian or Other Pacific Islander, Asian, other race, or multiracial.

** FPL is based on family income and family size and composition using the U.S. Census Bureau's poverty thresholds. Imputed income was used for persons aged 4 months–17 years without reported family income. https://www.census.gov/topics/income-poverty/poverty/guidance/poverty-measures.html

^††^ Special health care needs status is based on responses to the Children with Special Health Care Needs standardized five-item screener that included 1) the need for or use of medications (other than vitamins) prescribed by a doctor; 2) the need for or use of medical care, mental health, or educational services beyond those of a similarly aged person (referred to as “average use”); 3) limitation in the ability to do things most persons of the same age can do; 4) the need for or use of specialized therapies such as physical, occupational, or speech therapy; and 5) the need for or receipt of treatment or counseling for an emotional, behavioral, or developmental problem. https://www.census.gov/content/dam/Census/programs-surveys/nsch/tech-documentation/methodology/2016-NSCH-Methodology-Report.pdf; https://www.census.gov/content/dam/Census/programs-surveys/nsch/tech-documentation/methodology/2017-NSCH-Methodology-Report.pdf; https://www2.census.gov/programs-surveys/nsch/technical-documentation/methodology/2018-NSCH-Methodology-Report.pdf

^§§^ Regular bedtime is defined as a response of “always” to the question about how often the person aged 4 months–17 years goes to bed at approximately the same time on weeknights.

State-level prevalence of short sleep duration overall ranged from 25.3% in Maine to 48.9% in Mississippi (Table 2). States with the highest prevalence of short sleep duration were concentrated in the Southeast (Figure).

**FIGURE:**
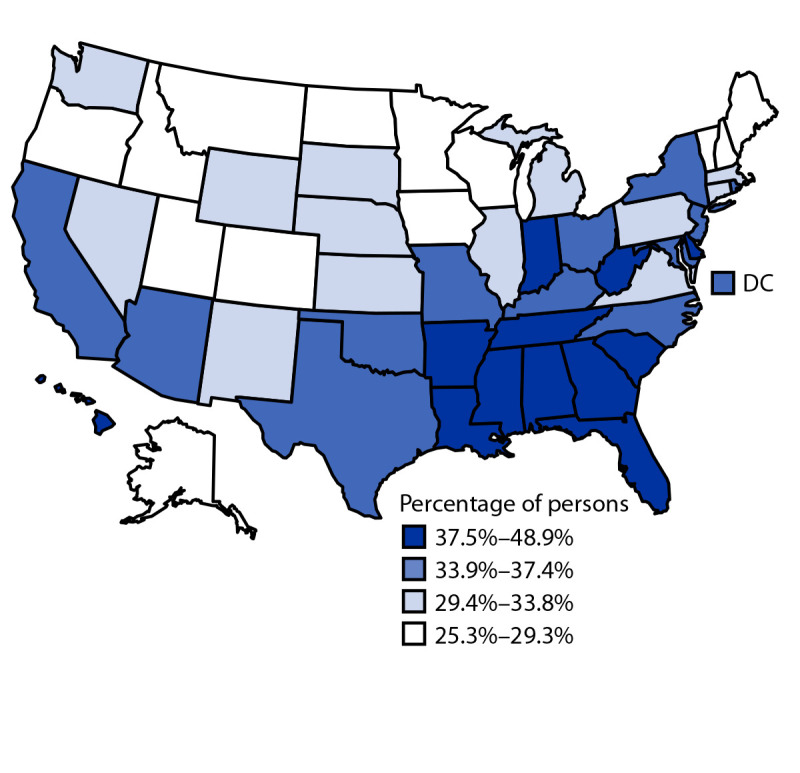
Prevalence of short sleep duration* among persons aged 4 months–17 years, by state — National Survey of Children’s Health, United States, 2016–2018 **Abbreviation:** DC = District of Columbia. * Short sleep duration is defined as <12 hours for infants aged 4–11 months, <11 hours for children aged 1–2 years, <10 hours for children aged 3–5 years, <9 hours for children aged 6–12 years, and <8 hours for adolescents aged 13–17 years.

## Discussion

During 2016–2018, approximately one third of persons aged 4 months–17 years (34.9%) got less sleep than is recommended for their age (*1*). Younger persons within this age group were particularly at risk for short sleep duration; the prevalence of short sleep duration decreased with age from infancy (40.3%) to adolescence (31.2%). Previous prevalence estimates of short sleep duration among adolescents were significantly higher: based on self-report from the 2015 Youth Risk Behavior Surveys,^†††^ nearly three quarters of high school students nationally and approximately one half of middle school students in nine states reported getting less sleep than recommended for their age (*2*). This difference might be explained by NSCH’s reliance on parent report rather than self-report with Youth Risk Behavior Surveys. Parents might overestimate the amount of sleep their older children or adolescents receive (*3*). However, one study that compared total sleep time based on parent report and child or adolescent report with that measured by a sleep study (i.e., polysomnography^§§§^) found that children and adolescents overestimated total sleep time to a lesser extent than did their parents, but the difference between child or adolescent and parent reports was small (*4*). Agreement between parent and child or adolescent report was similar for children aged 9–12 years and adolescents aged 13–17 years (*4*).

Before 2016, the NSCH did not ask parents about hours of sleep, but rather asked, “During the past week, on how many nights did [child] get enough sleep for a child his/her age?” Patterns in the prevalence of inadequate sleep (defined as not enough sleep ≥1 night during the past week) contrast with the current report; specifically, the prevalence of inadequate sleep was highest among adolescents aged 14–17 years and lowest among children aged 6–9 years (*5*). Trends based on parent education and household income also differed (*5*).

In the current study, short sleep duration was elevated among racial and ethnic minority groups, especially among Black persons aged 4 months–17 years, among whom approximately one half had short sleep duration. Short sleep duration was also more prevalent among families with lower income or lower parental educational attainment. In previous research, sleep disparity was associated with various social determinants of health (e.g., poverty, food insecurity, and perceived racism), which can increase chronic and acute stress and result in environmental and psychological factors that negatively affect sleep duration and can compound long-term health risks (*6*). Some parents, particularly those affected by socioeconomic and racial disparities, might face additional challenges to ensuring their infants, children, and adolescents get sufficient, quality sleep. For example, parents who work multiple jobs or do shift work might have difficulty implementing a consistent bedtime (*6*). In addition, a family’s housing situation could make achieving a quiet, comfortable sleep environment difficult because of noise, lack of sleeping space, or disruptive, unsafe, or violent neighborhoods (*6*).

The prevalence of short sleep duration was higher among persons aged 4 months–17 years whose current health was rated less positively and among those with special health care needs. Multiple conditions, including attention deficit/hyperactivity syndrome or other neurodevelopmental disorders, have been associated with sleep problems as well as sleep behaviors that might be amenable to behavioral intervention (*7*).

The prevalence of short sleep duration was highest in the Southeast, similar to geographic variation in adequate sleep observed for adults (*8*). This pattern might be partially explained by a higher prevalence of risks associated with poverty and racial and ethnic minority status in these states.^¶¶¶^ In a previous report of short sleep duration among high school students in 30 states, most of the states with a high prevalence of short sleep duration were in the Midwest and Northeast (*2*). However, that report relied on self-report rather than parent report, did not include younger children, and excluded 20 states.

The findings in this report are subject to at least five limitations. First, sleep duration was obtained by parent report without objective measures, such as actigraphy**** or polysomnography. Second, parent reports of sleep duration might be less reliable than are self-report for older children or adolescents (*3*). Third, responses might be affected by recall bias, interpretation of items, or social desirability. Fourth, the statistical weighting might not completely account for nonresponse bias. Finally, the analyses with race/ethnicity were univariate and did not adjust for other covarying sociodemographic characteristics.

Insufficient sleep is an important risk factor for poor physical and mental health in infants, children, and adolescents (*1*). Parents can help persons aged 4 months–17 years get the sleep they need by supporting good sleep habits. Establishing a regular bedtime is a good foundation and is associated with more sleep (*9*,*10*). The AASM’s Bedtime Calculator^††††^ identifies appropriate bedtimes based on age-specific sleep duration recommendations and provides tips on bedtime routines for parents of infants and children and for adolescents and adults. Clinicians and educators can guide parents about the importance of sleep at all ages and discuss sleep routines and sleep problems with parents, children, and adolescents, paying attention to those with special health care needs (*7*). When advising parents on how to improve their infant’s, child’s, or adolescent’s sleep, challenges that they might face because of their social and environmental context should be considered. School districts can support adequate sleep for adolescents by delaying school start times as recommended by several medical associations (*2*).

**Summary**

**What is already known about this topic?**

Infants, children, and adolescents who do not get sufficient sleep are at increased risk for adverse health outcomes. Most adolescents report sleeping less than the recommended amount. Little is known about sleep duration in infants and children.

**What is added by this report?**

During 2016–2018, approximately one third of children aged 4 months–17 years slept less than recommended for their age, particularly those from racial and ethnic minority groups, of low socioeconomic status, and with special health care needs. Infants, children, and adolescents with regular bedtimes were more likely to get enough sleep.

**What are the implications for public health practice?**

Public health practitioners, educators, and clinicians can advise parents about the importance of infants, children, and adolescents meeting recommended sleep durations, investigate the social and environmental context that affects sleep, and support parents in implementing consistent bedtimes.

## Footnotes

*
https://mchb.hrsa.gov/

† https://www.census.gov/content/dam/Census/programs-surveys/nsch/tech-documentation/methodology/2016-NSCH-Methodology-Report.pdf; https://www.census.gov/content/dam/Census/programs-surveys/nsch/tech-documentation/methodology/2017-NSCH-Methodology-Report.pdf; https://www2.census.gov/programs-surveys/nsch/technical-documentation/methodology/2018-NSCH-Methodology-Report.pdf

§ Response options were “less than 7 hours,” “7 hours,” “8 hours,” “9 hours,” “10 hours,” “11 hours,” and “12 or more hours.”

¶ The question changed slightly in 2018, when parents were asked, “During the past week, how many hours of sleep did this child get on most weeknights?” Response options for 2016–2018 were “less than 6 hours,” “6 hours,” “7 hours,” “8 hours,” “9 hours,” “10 hours,” and “11 or more hours.”

** Response options were “always,” “usually,” “sometimes,” “rarely,” and “never.”

†† AASM did not include recommendations for persons aged <4 months; therefore, short sleep duration was not assessed for this age group.

§§
https://www.census.gov/content/dam/Census/programs-surveys/nsch/tech-documentation/methodology/2016-NSCH-Methodology-Report.pdf

¶¶ Weighted estimates were used to generalize to state and national resident populations.

†††
https://www.cdc.gov/healthyyouth/data/yrbs/index.htm

§§§
https://www.nhlbi.nih.gov/health-topics/sleep-studies

¶¶¶
https://www.census.gov/library/visualizations/interactive.html

****
https://sleepeducation.org/patients/actigraphy/

††††
https://sleepeducation.org/healthy-sleep/bedtime-calculator/
